# BAT117213: Ileal bile acid transporter (IBAT) inhibition as a treatment for pruritus in primary biliary cirrhosis: study protocol for a randomised controlled trial

**DOI:** 10.1186/s12876-016-0481-9

**Published:** 2016-07-19

**Authors:** Vinod S. Hegade, Stuart F. W. Kendrick, Robert L. Dobbins, Sam R. Miller, Duncan Richards, James Storey, George Dukes, Kim Gilchrist, Susan Vallow, Graeme J. Alexander, Margaret Corrigan, Gideon M. Hirschfield, David E. J. Jones

**Affiliations:** NIHR Newcastle Biomedical Research Centre and Institute of Cellular Medicine, Newcastle University, Newcastle upon Tyne, UK; Institute of Cellular Medicine, William Leech Building, Framlington Place, Newcastle University, Newcastle upon Tyne, NE2 4HH UK; GlaxoSmithKline Research and Development, Medicines Research Centre, Stevenage, UK; GlaxoSmithKline, Research Triangle Park, NC USA; GlaxoSmithKline, Collegeville, PA USA; Department of Hepatology, Cambridge University Hospitals NHS Foundation Trust Cambridge, Cambridge, UK; Centre for Liver Research, University of Birmingham and NIHR Birmingham Liver Biomedical Research Unit, Birmingham, UK

**Keywords:** Pruritus, Primary biliary cholangitis, PBC, Ileal bile acid transporter, IBAT

## Abstract

**Background:**

Pruritus (itch) is a symptom commonly experienced by patients with cholestatic liver diseases such as primary biliary cholangitis (PBC, previously referred to as primary biliary cirrhosis). Bile acids (BAs) have been proposed as potential pruritogens in PBC. The ileal bile acid transporter (IBAT) protein expressed in the distal ileum plays a key role in the enterohepatic circulation of BAs. Pharmacological inhibition of IBAT with GSK2330672 may reduce BA levels in the systemic circulation and improve pruritus.

**Methods:**

This clinical study (BAT117213 study) is sponsored by GlaxoSmithKline (GSK) with associated exploratory studies supported by the National Institute for Health Research (NIHR). It is a phase 2a, multi-centre, randomised, double bind, placebo controlled, cross-over trial for PBC patients with pruritus. The primary objective is to investigate the safety and tolerability of repeat doses of GSK2330672, and explore whether GSK2330672 administration for 14 days improves pruritus compared with placebo. The key outcomes include improvement in pruritus scores evaluated on a numerical rating scale and other PBC symptoms in an electronic diary completed twice daily by the patients. The secondary outcomes include the evaluation of the effect of GSK2330672 on total serum bile acid (BA) concentrations, serum markers of BA synthesis and steady-state pharmacokinetics of ursodeoxycholic acid (UDCA).

**Discussion:**

BAT117213 study is the first randomised controlled crossover trial of ileal bile acid transporter inhibitor, a novel class of drug to treat pruritus in PBC. The main strengths of the trial are utility of a novel, study specific, electronic symptom diary as patient reported outcome to measure the treatment response objectively and the crossover design that allows estimating the treatment effect in a smaller number of patients. The outcome of this trial will inform the trial design of future development phase of the IBAT inhibitor drug. The trial will also provide opportunity to conduct metabonomic and gut microbiome studies as explorative and mechanistic research in patients with cholestatic pruritus.

**Trial registration:**

EudraCT number: 2012-005531-84, ClinicalTrials.gov Identifier: NCT01899703, registered on 3^rd^ July 2013

## Background

Primary biliary cholangitis (cirrhosis) (PBC) is an autoimmune chronic cholestatic liver disease with a prevalence of 30/100,000, typically affecting middle aged women (female: male ratio 10:1) [1]. In untreated cases immunologically mediated chronic cholestasis ultimately results in liver cirrhosis with associated complications such as portal hypertension, varices, ascites, hepatocellular carcinoma and death. The precise aetiology of PBC is unclear, although genetic and environmental factors are thought to play a key role.

Pruritus (itch) is one of the characteristic symptoms of PBC and can affect patients at any stage of the disease [2]. Recently, we studied the scale of the pruritus symptom within the United Kingdom (UK)-PBC cohort, a national cohort of over 3000 PBC patients recruited from every hospital in the UK. In this cohort 60–70 % of PBC patients reported experience of pruritus at some point in the course of the disease, 30 % had persistent pruritus and 15 % suffered with severe pruritus since the diagnosis of PBC [3]. A similar scale of symptom burden has also been reported in PBC cohorts from USA and Italy [4]. Pruritus has a negative impact on perceived quality of life in PBC patients and has been associated with sleep deprivation, worsened day time fatigue and when severe, may lead to depression and suicidal tendencies [5].

Ursodeoxycholic acid (UDCA), the current standard of care for PBC patients and the only licenced therapy for PBC has no role in treating pruritus [2]. Current treatment of pruritus in PBC involves step-wise use of specific anti-pruritic agents in line with current international guidelines [2, 6]. These drugs include cholestyramine, rifampicin, naltrexone and sertraline. Of these, cholestyramine is the only licensed drug for treatment of cholestatic pruritus and use of other drugs is “off-label”. The limitations of these drugs are that their efficacy is not universal, treatment is often associated with side effects and there is a need for regular monitoring for liver toxicity. Patients with medically refractory pruritus may either need to undergo phototherapy, invasive interventions such as nasobiliary drainage or extracorporeal albumin dialysis for temporary relief of pruritus, or may be considered for liver transplantation (LT) which is typically curative. Therefore, development of better drug therapies with fewer side effects is an unmet clinical need for PBC patients [7].

### Ileal bile acid transporter (IBAT)

Primary BAs are synthesized in the liver from an enzymatic catabolism of cholesterol, a process regulated by enzyme cytochrome P450 (CYP) 7A1. Unconjugated BAs are conjugated in hepatocytes with glycine and taurine, secreted into the bile and stored in the gallbladder. Upon ingestion of a meal, conjugated BAs (“bile salts”) are released into the intestinal lumen where they facilitate absorption of fat and fat soluble vitamins. After their normal physiological function is completed in the intestine, BAs reach the ileum where they are reabsorbed. The ileal bile acid transporter [(IBAT), also called apical sodium dependent bile acid transporter (ASBT)], is a protein predominantly located in the terminal ileum and serves as the main transporter mediating the ileal uptake of conjugated BAs and their return to the liver via the portal circulation (enterohepatic circulation) [8].

Bile salts (and their protonated form, BAs) have been suggested to play role in the pathogenesis of pruritus in cholestatic conditions. In cholestasis, the ileal uptake of BAs has been shown to be upregulated [9]. Also, the evidence that pruritus dramatically improves in patients undergoing nasobiliary drainage [10] and is effectively cured by LT [11] suggests a direct or indirect role for BAs in mediating cholestatic pruritus. Therefore a pharmaceutical agent that can reduce their levels in the enterohepatic and systemic circulations may be predicted to improve pruritus. In two animal studies treatment with IBAT inhibitors SC-435 and A4250 produced BA malabsorption and attenuated BA-mediated cholestatic liver injury by reducing biliary BA output [12, 13]. In humans, use of IBAT inhibitor A4250 has been shown to decrease the serum BAs and increase faecal BAs by highly efficient interruption of their enterohepatic circulation with no serious adverse events [14].

### GSK2330672

**Fig. 1:**
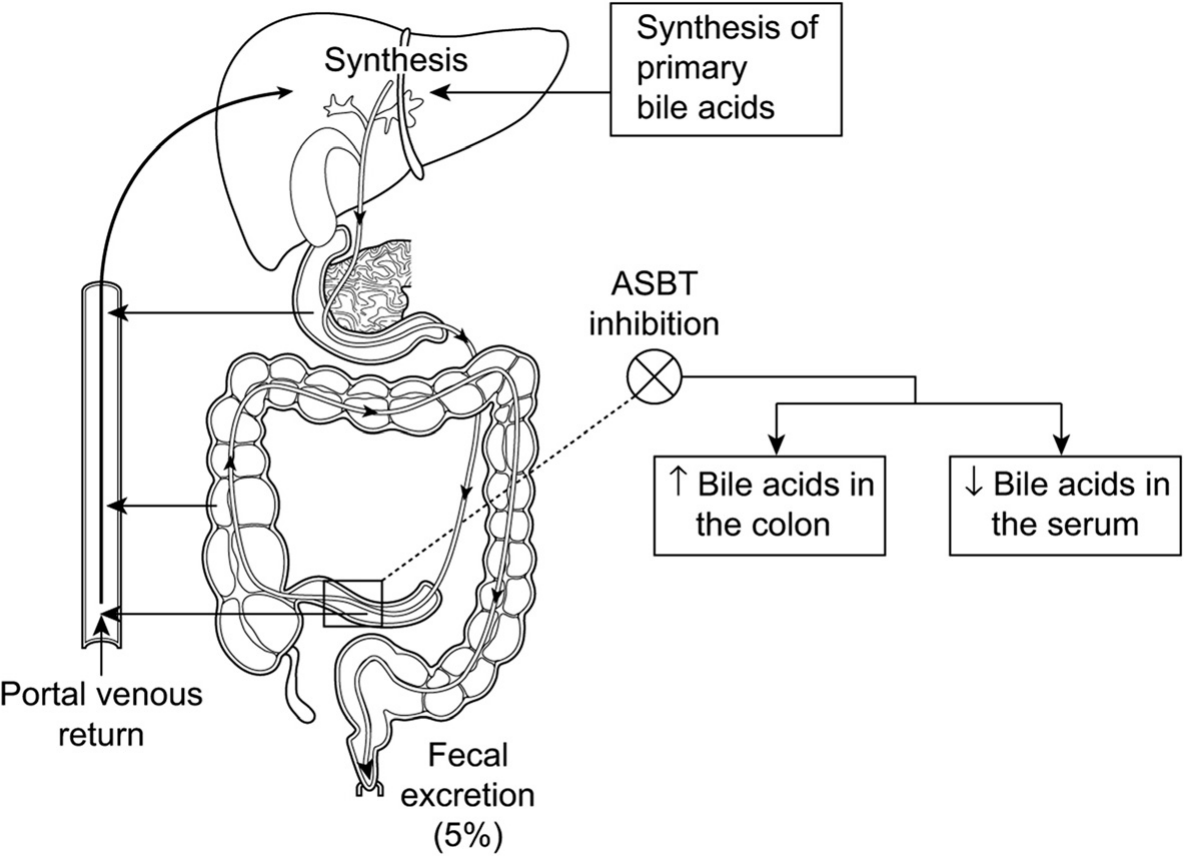
Mechanism of IBAT (ASBT) inhibitor drug. GSK2330672 interrupts the enterohepatic circulation of bile acids by selectively inhibiting the IBAT protein located in the terminal ileum, thereby reducing the levels of bile acids in the systemic circulation. (Image reproduced with permission from [31]). IBAT, ileal bile acid transporter; ASBT, apical sodium-dependent bile acid transporter

GSK2330672 is a selective inhibitor of human IBAT and it is designed to be a non-absorbable agent restricted to the gastrointestinal (GI) tract. GSK2330672 is expected to block the uptake of BAs in the terminal ileum, increase their excretion in the faeces and decrease the amount of BAs returning to the liver via enterohepatic circulation (Fig. 1). Therefore treatment of PBC patients with oral GSK2330672 is postulated to reduce concentrations of BAs in the systemic circulation and in turn improve pruritus.

In phase I studies involving 42 healthy volunteers single and repeat doses of GSK2330672 for 12 days were shown to be safe and tolerable (ClinicalTrials.gov Identifier: NCT01416324). GI symptoms were the most common reported drug-related adverse events (AEs). These included diarrhoea, abdominal pain, bowel movement irregularity and positive faecal occult blood tests. All AEs were considered mild or moderate in severity.

## Methods

### Study design and overview

The BAT117213 study is a Phase 2a trial, designed to investigate treatment with GSK2330672 in PBC patients with pruritus (ClinicalTrials.gov Identifier: NCT01899703). This is a multicentre, randomized, double-blind, placebo-controlled, two-period cross-over trial which in addition to studying the safety and efficacy of the drug will provide an opportunity to conduct explorative studies (including metabonomic and microbiomic studies) to develop novel mechanistic insights into cholestatic pruritus.

**Fig. 2:**
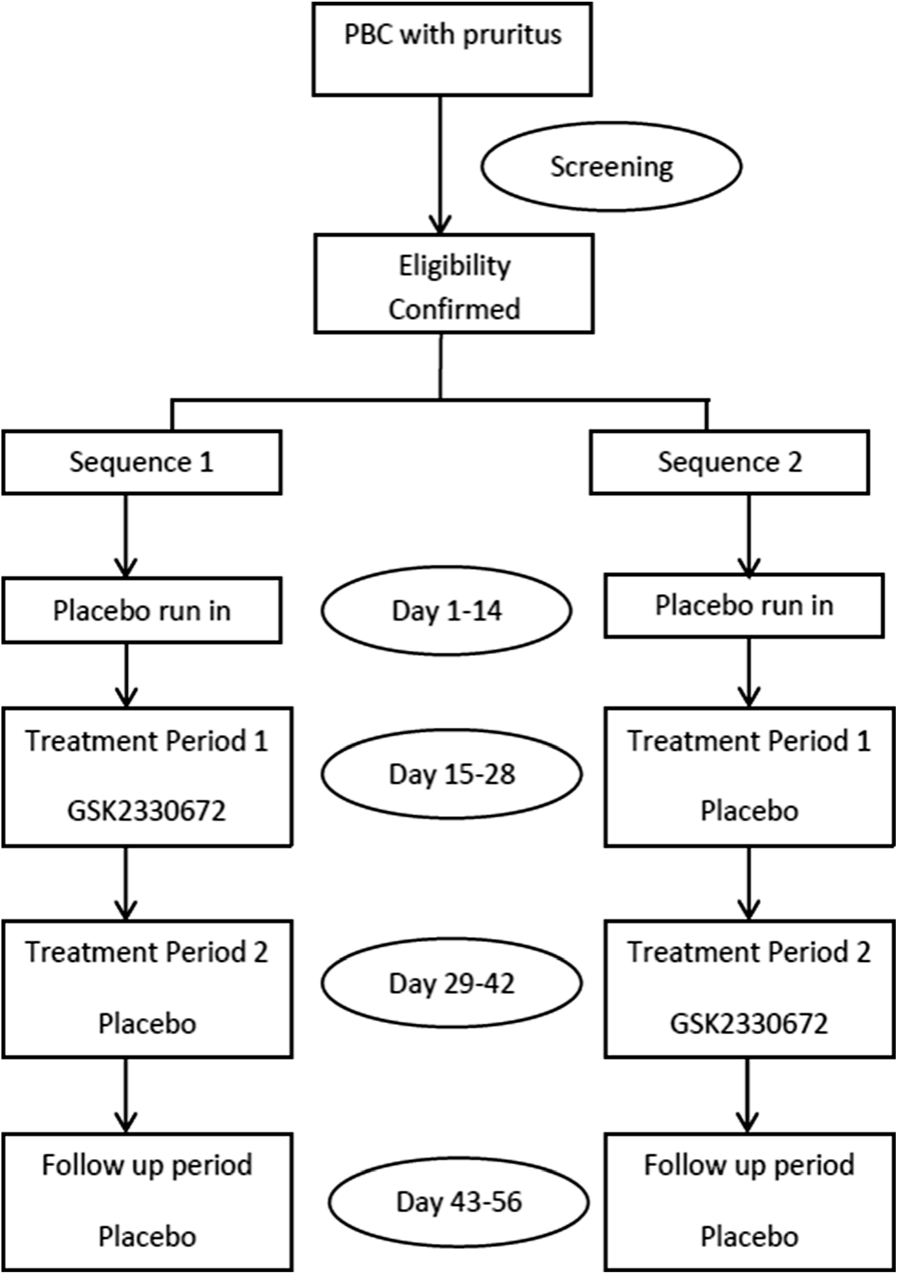
Flowchart of BAT117213 study design

Following written informed consent, patients with PBC and pruritus were screened to establish study eligibility. Eligible subjects participated in a two-week placebo run-in period followed by randomization in a crossover fashion to receive placebo or GSK2330672 treatment during two consecutive two-week study periods (Sequence 1/Sequence 2) (Fig. 2). Subjects then participated in a two-week follow up period of placebo dosing. Total duration of the study was 56 days from the first day of dosing.

### Study population

The study population consisted of PBC patients with ongoing pruritus. All participants had a diagnosis of definite or probable PBC established according to recognised criteria [2, 6]. Key inclusion and exclusion criteria for study eligibility are detailed in Table 1. The trial entry criteria for ongoing pruritus was defined as: i) severe pruritus significantly impacting daily life and proven refractory to medical therapy, or ii) pruritus that is newly diagnosed or untreated, or iii) pruritus that is unresolved with the use of a single antipruritic agent. To determine subject eligibility for study enrolment outpatient screening was performed within 45 days before the first dose administration. Subjects meeting all the inclusion criteria and no exclusion criteria were enrolled by a designated investigator from the centre.

**Table 1:** Eligibility criteria

A subject will be eligible for inclusion in this study only if all of the following criteria apply: 1. Male or female aged between 18 and 75 years of age inclusive, at the time of signing the informed consent. 2. Proven or likely PBC, as demonstrated by the patient presenting with at least 2 of the following: • History of sustained increased AP levels first recognized at least 6 months prior to Day 1 • Positive AMA titer (>1:40 titer on immunofluorescence or M2 positive by ELISA) or PBC-specific antinuclear antibodies (antinuclear dot and nuclear rim positive) • Liver biopsy consistent with PBC. 3. Screening AP value < 10 × ULN. 4. Subjects should be on stable doses of UDCA for >8 weeks at time of screening. Subjects not taking UDCA due to intolerance may be enrolled into this study following agreement by the GSK medical monitor. 5. Symptoms of pruritus as follows (one of the following): • PBC patients with severe symptoms of pruritus that significantly impact daily life and have proven refractory after at least one previous therapy has been discontinued due to inadequate clinical response, poor tolerability or adverse events. Temporary response to cooling, 1 % menthol in aqueous cream, nasobiliary drainage or MARS therapy is still compatible with refractory itch. • PBC patients with unresolved symptoms with use of a single antipruritic agent who can tolerate washout of current therapy for the duration of the trial. • PBC patients seeking treatment for pruritus that is newly diagnosed or previously untreated. 6. A female subject is eligible to participate if she is not pregnant, as confirmed by a negative serum human chorionic gonadotropin (hCG) test or at least one of the following conditions applies: • Non-reproductive potential defined as pre-menopausal females with a documented tubal ligation or hysterectomy; or postmenopausal defined as 12 months of spontaneous amenorrhea • Females on hormone replacement therapy (HRT) and whose menopausal status is in doubt will be required to use one of the highly effective contraception methods along with either a second form of highly effective contraception or barrier protection (condoms with spermicide) if they wish to continue their HRT during the study. Otherwise, they must discontinue HRT to allow confirmation of post-menopausal status prior to study enrolment. • Reproductive potential and agrees to follow one of the specified contraception options for the specified duration of time. 7. Capable of giving written informed consent, which includes compliance with the requirements and restrictions listed in the consent form. Main exclusion criteria: 1. Screening total bilirubin >1.5x ULN. Isolated bilirubin >1.5xULN is acceptable if bilirubin is fractionated and direct bilirubin <35 %. 2. Screening ALT or AST >4x ULN. 3. Screening serum creatinine >2.5 mg/dL (221 umol/L). 4. History or presence of hepatic decompensation (e.g., variceal bleeds, encephalopathy, or poorly controlled ascites). 5. History or presence of other concomitant liver diseases including hepatitis due to hepatitis B or C virus (HCV, HBV) infection, primary sclerosing cholangitis (PSC), alcoholic liver disease, definite autoimmune hepatitis or biopsy proven non-alcoholic steatohepatitis (NASH). 6. Administration of the following drugs at any time during the 3 months prior to screening for the study: colchicine, methotrexate, azathioprine, or systemic corticosteroids. 7. Current or chronic history of inflammatory bowel disease, chronic diarrhoea, Crohn’s disease or diarrhoea related to malabsorption syndromes. 8. Faecal occult blood positive test at screening. 9. Based on averaged QTc values of triplicate ECGs obtained at least 5 min apart: • QTc ≥ 450 msec; or • QTc ≥ 480 msec in subjects with Bundle Branch Block. 10. History of sensitivity to heparin or heparin-induced thrombocytopenia. 11. History of sensitivity to any of the study medications, or components thereof or a history of drug or other allergy that, in the opinion of the investigator or GSK Medical Monitor, contraindicates their participation. 12. History of regular alcohol consumption within 6 months of the study defined as an average weekly intake of >21 units for males or >14 units for females. 13. A positive pre-study drug/alcohol screen. A minimum list of drugs that will be screened for include amphetamines, barbiturates, cocaine, opiates, cannabinoids and benzodiazepines. 14. Where participation in the study would result in donation of blood or blood products in excess of 500 mL within a 56 day period. 15. The subject has participated in a clinical trial and has received an investigational product within the following time period prior to the first dosing day in the current study: 30 days, 5 half-lives or twice the duration of the biological effect of the investigational product (whichever is longer). 16. Exposure to more than four new chemical entities within 12 months prior to the first dosing day.	

### Study objectives and outcomes

The primary objective of this trial is to investigate the safety and tolerability of oral GSK2330672 compared with placebo when administered for 14 days to PBC patients treated with UDCA. The secondary objectives are: 1) to evaluate the effects of oral GSK2330672 on subjects’ experience of pruritus and its impact; 2) to demonstrate the lack of effect of oral GSK2330672 on steady-state pharmacokinetics (PK) of UDCA when UDCA is administered alone or in combination with GSK2330672; 3) to investigate the steady state PK of oral GSK2330672; 4) to evaluate the effects of oral GSK2330672 on total serum BA concentrations and serum markers of BA synthesis [7-alpha-hydroxy-4-cholesten-3-one (C4)]. Exploratory objectives of the study include investigating effects of 14-day oral administration of GSK2330672 on markers of disease progression, subject’s experience of benefits and disadvantages with GSK2330672, metabonomics, microbiomics and pharmacogenomics. The primary, secondary and exploratory outcome measures are given in Table 2.

**Table 2:** Primary, secondary and exploratory outcome measures of the BAT117213 study

1. Primary outcome measures: • Safety assessment following repeat doses of oral GSK2330672 Safety will be assessed using clinical haematology, clinical chemistry, urinalysis, single 12-lead electrocardiograms (ECGs), vital sign measurements including systolic and diastolic blood pressure (BP) and pulse rate. • Tolerability assessment using Gastrointestinal Symptom Rating Scale (GSRS) Subjects will be asked to complete GSRS, a validated scale and the scale will be used to assess symptoms experienced by subject over the preceding 5 to 7 days • Faecal occult blood (FOB) testing FOB monitoring for symptomatic or visible gastrointestinal bleeding or asymptomatic occult bleeding 2. Secondary outcome measures: • Subject reported outcomes-daily pruritus 0 to 10 point scale This scale will be implemented to measure symptoms of itching as well as other associated symptoms twice daily in the morning and evening (approximately the time of drug dosing). The severity of itching symptoms from “0” (no itching) to “10” (worst possible itching) will be recorded • Subject reported outcomes-5D-itch scale The 5-D itch scale covers five dimensions of itching experienced by subjects including duration, degree, direction, disability and distribution • Subject reported outcomes-PBC-40 quality of life (QoL) scale The PBC-40 QoL scale has six domains; cognitive, itch, fatigue, social, emotional and (other) symptoms • Measurement of serum profiles of total bile acid concentrations and 7-alpha hydroxy-4-cholesten-3-one (C4). C4 is the first committed step of bile acid synthesis from cholesterol • Steady-state pharmacokinetics (PK) assessment of UDCA and its taurine and glycine conjugates taurodeoxycholic acid (TUDCA) and glycoursodeoxcholic acid (GUDCA). Blood sample will be collected for measurements of steady state PK parameters of UDCA and its metabolites including maximum observed plasma concentration (C_max_), time to C_max_ (t_max_) and terminal phase half-life (t1/2). 3. Exploratory outcome measures: • Markers of disease progression: ALT/AST, AP, GGT, bilirubin, albumin, PT/INR • An exit interview conducted at end of follow-up phase to assess subject’s experience of benefits and disadvantages with GSK2330672 • Pharmacogenomics for genes related to pruritus and GSK2330672 response • Metabonomics to study serum bile acid species, serum autotaxin and FGF-19 before and after treatment with GSK2330672 • Microbiomics to study gut microbiota in PBC patients with pruritus	

### Recruitment and consent

The study is a UK multicentre study and recruitment was planned in three large, tertiary referral National Health Service (NHS) hospitals based in Newcastle, Birmingham and Cambridge. Patients were recruited from the out-patient department cohorts of these hospitals and in addition, trial information was published in newsletters and magazines from the UK-PBC research group and patient support groups (LIVErNORTH and PBC Foundation). Any PBC patient interested in participating in the study could contact the study team at the centre nearest to their location either directly or via referral from local primary or secondary care physicians. The UK-PBC platform was utilised for recruitment using a similar approach to the to the RIT-PBC trial reported recently by our group [15]. The established UK-PBC database was screened for patients with PBC-40 itch domain scores meeting the definitions of persistent and/or severe pruritus. The clinicians looking after these patients were contacted to approach the patients and interested patients were referred to their local recruiting centre. All participants gave their written consent to participation before screening investigations were performed. Participants completed the consent process with study investigators trained in Good Clinical Practice (GCP) and assessment of capacity.

### Randomisation

All eligible subjects enrolled in the study were randomised to either Sequence 1 or Sequence 2 to receive oral placebo or GSK2330672 for a 14-day period in a cross-over fashion (Fig. 2). Sequence 1 was GSK2330672 for 14 days followed by placebo for 14 days and Sequence 2 was placebo for 14 days followed by GSK2330672 for 14 days. Randomisation was carried out via a dedicated electronic system for randomisation-RAMOS (Randomisation and Medication Ordering System) by generating a unique randomisation number for each participant that linked to the corresponding allocated sequence of study drug.

### Study treatment

The investigational medicinal product used in this study was GSK2330672. The control intervention was placebo. Both GSK233072 and placebo were manufactured at a dedicated manufacturing unit in London (UK) and dispensed as 30 g aliquots of oral solution into amber glass bottles for distribution to participating study centres. The study centres supplied solutions to subjects in accordance with the randomization schedule. Subjects consumed the entire quantity of one or two bottles of study drug twice daily followed by two 50 mL rinses of water. All patients started the study with 14-days placebo run in period followed by 14-days treatment with GSK2330672 or placebo in a cross over fashion.

### Dose escalation and stopping criteria

The initial dose of GSK2330672 was 45 mg and all patients were asked to increase the dose to 90 mg on day 4. If this was not tolerated, they were asked to continue at 45 mg and attempt a dose increase again two days later. If 90 mg could not be tolerated by the end of day 7, subjects were asked to continue only 45 mg.

Following stopping criteria were in place to assure subject safety: 1) to stop the study treatment if the stopping criteria for liver chemistry were met [ALT >5-8 x upper limit of normal (ULN), bilirubin > 1.5-2 x ULN], and 2) to withdraw the subject from the study if corrected QT (QTc) interval withdrawal criteria were met based on their average values on triplicate ECGs separated by five minutes. These were QTc > 500 msec, or uncorrected QT >600 msec, or QTc >60 msec change from baseline). If a subject met the stopping criteria, appropriate safety follow-up assessments and procedures were completed.

### Concomitant medications

Before starting the study, all patients were advised to stop using their usual anti-pruritic agents including cholestyramine, colesevelam, rifampicin, naltrexone, sertraline, gabapentin and anti-histamines. The use of these medications was prohibited during the study period until the final follow-up period when rescue medications were permitted. Application of topical agents used to relieve pruritus was permitted during the study only if agents did not contain active ingredients in the list of prohibited agents and with prior agreement of the clinical investigator. Subjects were asked to abstain from taking new prescription or new non-prescription drugs (including vitamins and dietary or herbal supplements), from the start of the placebo run-in period until completion of the follow-up visit. The use of UDCA was permitted and patients who were on UDCA were standardised to receive Ursofalk®(Dr. Falk Pharma UK Ltd) once daily preparation at dose 13–15 mg/kg/day and instructed to take it at bed time.

### Patient reported outcomes

Existing patient reported outcome (PRO) measures to assess the impact of PBC symptoms include the PBC-40, a widely acceptable, validated, disease-specific questionnaire and the 5-D Itch scale [16, 17]. However, for this study a more specific PRO measure was needed that could detect the severity and variability of pruritus and other PBC symptoms and potential treatment effects on a daily basis with a short recall period. The development of such a measure began with interviews with PBC patients to identify additional characteristics of pruritus and other symptoms and their impact on sleep and daily activities. With input from PBC patients and PRO experts a new electronic patient reported outcome (ePRO) diary was developed to assess the severity of the pruritus and other PBC symptoms. Subjects completed the ePRO diary every morning and evening before dosing the study drug. In the ePRO diary pruritus severity was rated using a numerical rating scale (NRS). Psychometric testing to support the validity and reliability of the ePRO will be evaluated with data from the current clinical study.

## Data analysis

### Statistical analysis

This trial is designed to estimate the effect of study drug GSK2330672 relative to placebo when co-administered with UDCA on pruritus symptom, markers of efficacy and disease progression and the PK of UDCA. No formal hypothesis will be tested.

The efficacy endpoint in this study is the patient reported rating of pruritus severity scores. Pruritus will be measured in three different PROs: pruritus NRS using the ePRO, the 5-D itch scale and the PBC-40 questionnaire [16, 17]. Changes in pruritus NRS will be used as the key measure of the efficacy endpoint and will be analysed using a mixed effects model with fixed effect terms for treatment period and sequence to examine differences between GSK2330672 and placebo. Subject will be treated as a random effect in the model. Point estimates and their associated 95 % confidence interval (CI) will be constructed for the mean differences in pruritus severity scores.

Data from subjects that are co-administered UDCA as part of their standard care will be analysed similarly for PK endpoints. Following log-transformation, maximum observed plasma concentration (C_max_), AUC (0–12 h) and AUC (12–24 h) of UDCA and glycine and taurine conjugated metabolites of UDCA (TUDCA and GUDCA) will be separately analysed. This will be done using a mixed effects model with fixed effect terms for treatment period and sequence to examine differences between UDCA administration with and without GSK2330672. Point estimates and corresponding 90 % CI will also be constructed for the difference and/or ratio between the mean of the test treatment (UDCA plus GSK2330672) and the mean of the reference treatment (UDCA alone).

### Sample size

The efficacy endpoint in this study is pruritus score and the sample size for efficacy endpoint is based on the pruritus 0 to 10 points scale. On this scale the average effect of rifampicin is 1.62 points and the reported pooled total standard deviations of various anti-pruritic drugs ranges from 1.22 to 3.84 points [18, 19]. Assuming that GSK2330672 is at least as effective as rifampicin, a sample size of 40 will result in a reasonable power (>90 %) if the standard deviation (SD) is 3.1 points or less. For estimation of relative bioavailability 20 subjects taking UDCA are required to ensure that the resultant 90 % CI of the ratio will be within 0.8 and 1.25 assuming that the true ratio is 1 and the SD on the log10 scale is less than 0.25.

An initial sample-size of 40 subjects was selected based on considerations of both efficacy and PK endpoints. However, due to the uncertainty around sample-size assumptions a series of interim analyses for futility and possible sample-size re-estimation were carried out at regular intervals. Data from completed patients were reviewed by an unblinded review committee (composed of GSK personnel not directly involved in study conduct). As the probability of demonstrating sufficient difference was high, the sponsor revised the sample-size from 40 to 22. No other changes to study conduct were planned as a result of the interim analyses.

## Conduct of the trial

The conduct of the trial followed the principles outlined in the NHS research governance framework for health and social care, GCP and the guiding principles of the 2008 Declaration of Helsinki. The trial involved the participant visiting the study centre a total of six times including screening visit, day 1 visit, three consecutive fortnightly in-patient stays (each up to 36 h) and a follow up visit. The schedule of study procedures during these visits and data collection is summarised in Table 3.

Protocol deviation or exemptions were not allowed with the exception of immediate safety concerns. All Investigators at recruiting sites followed standard operative procedures for collection, handling, processing and storage of samples (blood, urine and stool) collected at study visits. All clinical and non-clinical subject data including medical history (to capture co-morbidities and concomitant medications) and physical examinations were entered into electronic case report forms (eCRFs). No patient identifiable information was entered in the eCRFs. All participants were allocated a unique study identifier which was used on eCRFs transmitted electronically to the sponsor and combined with data provided from other sources in a validated data system.

**Table 3:** BAT117213 study: schedule of procedures and data collection

Period description	Screening	Placebo run-in				Treatment period 1				Treatment period 2				Follow-up^f^			
Day (relative to Day 1)	-45 to-1 days	1	2–12	13	14	15	16–26	27	28	29	30–40	41	42	43	44	45–55	56
Admission to Unit				X				X				X					
Discharge						X				X				X			
Outpatient visit	X	X															X
Screening assessments^a^	X																
Brief Physical		X															X
12-lead ECG^b^	X	X			X				X				X				X
Vital signs	X	X			X				X				X				X
Urine drug/alcohol screen	X	X		X				X				X					X
β-hcg (women)	X	X		X				X				X					X
standard blood tests and urinalysis	X				X				X				X				X
Randomisation	X																
Study treatment dosing		X	X	X	X	X	X	X	X	X	X	X	X	X	X	X	
Concomitant medication review		X		X				X				X					X
Meal served				X	X	X		X	X	X		X	X	X			
Blood samples^c^					X				X				X				
Metabonomics (Stool and urine)				<-------------------->				<-------------------->				<----------------->					
Microbiomics (Stool)				<-------------------->				<-------------------->				<----------------->					
PBC-40 questionnaire^d^		X		X				X				X					X
GSRS^d^		X		X				X				X					X
5-D Itch scale^d^		X		X				X				X					X
Faecal Occult blood	X				X				X				X				X
Pruritus 0–10 point scale (electronic diary)^e^		<--------------------------------------------------------------------->															
AE assessment		<--------------------------------------------------------------------->															
PGx	For subjects who consent only. Collect one PGx sample after the start of dosing, preferably on day 1																
Exit interview																	X

^a^Screening assessments: informed consent for the study and PGX; demographics; complete physical; medical/medication/drug/alcohol history; Hepatitis B and Hepatitis C screen

^b^Single ECG to be performed, with the exception of screening and day 14 when this will be in triplicate

^c^Blood samples for GSK2330672, UDCA, bile acids, bile acid species, C4, Autotaxin, FGF19 and metabonomics

^d^patient reported outcome (PRO) assessments. On days in which PRO assessments are administered at study visits they should be administered before any other study procedure

^e^symptoms recorded twice daily (pre-dose of study drug)

^f^Rescue treatment with antipruritic agents can be instituted in subjects with severe itching during the placebo follow up period

### Study monitoring

The study sponsor performed periodic monitoring at each study centre to monitor the study conduct and site activity. The monitor had direct access to all relevant documents to verify the data for completeness, accuracy and authenticity and the site’s compliance with study protocol. All monitoring findings were reported and followed up in a timely manner. Periodic interim analysis of the trial were undertaken to determine as to whether the study should be modified, continued or terminated.

### Adverse events

AEs and serious adverse events (SAEs) were collected from the start of the placebo run-in period (day 1) until the follow-up contact (day 56). The investigator and site staff were responsible for detecting, documenting and reporting events that meet the definition of an AE or SAE. All SAEs were recorded and reported to the study sponsor within 24 h. Periodic reviews of the safety data were performed and presented during interim analysis to both the sponsor and the study investigators.

### Sponsorship, insurance and indemnity

In accordance with the Association of the British Pharmaceutical Industry (ABPI) guidance, the trial sponsor had policies in place regarding compensation for any trial related harm due to negligence or otherwise. The trial sponsor had insurance to cover indemnity in respect of potential liability arising from negligent harm related to study design. Due to the commercial nature of the study there were also arrangements for non-negligent compensation. The participating study centres were NHS hospitals and the NHS indemnity covered NHS staff and medical academic staff with honorary NHS contracts conducting the study for potential liability in respect of negligent harm arising from the conduct of the study.

### Trial status

The BAT117213 study was opened for recruitment in January 2014 with first patient recruited in March 2014. The initial recruitment target was 40 subjects. Following review of safety and efficacy of data from 11 patients at the first interim analysis in March 2015, the sponsor decided to continue the study recruitment. A second interim analysis of the data from 19 patients was performed in July 2015 and the sponsor decided to reduce the total sample size to 22 patients. The recruitment ended in October 2015 with all 22 patients randomised from two trial sites (Newcastle 13; Birmingham 9). The treatment follow-up of participants was completed in December 2015. The analysis of study data is currently ongoing and results are scheduled to be available in November 2016.

## Discussion

### Need for novel anti-pruritic drugs in PBC

Pruritus is a complex symptom and the drug treatment of pruritus in PBC patients remains a challenge in clinical practice. The four main classes of drugs that are recommended by current guidelines [2, 6] include bile acid sequestrants (cholestyramine), enzyme inducers (rifampicin), opioid antagonists (naltrexone) and selective serotonin re-uptake inhibitors (sertraline). These drugs are limited by their lack of universal efficacy, poor compliance (especially cholestyramine) and the need for regular monitoring for liver toxicity (rifampicin). Cholestyramine and rifampicin have good reports but clinical experience of both naltrexone and sertraline has been disappointing for many clinicians [2].

A critical review of literature shows that the strength of evidence for current anti-pruritic drug therapy is poor. Cholestyramine, the current first-line therapy was last studied over five decades ago but has never been subjected to randomised placebo-controlled trials and has evidence category II-2 (cohort or case control analytical studies) [20–24]. Only rifampicin and naltrexone have been studied in controlled trials [18, 19, 25–27] and sertraline (evidence category II-2) is the last agent investigated with a positive outcome on pruritus [28]. A number of other drugs have been investigated but with little success and more recently both gabapentin (2006) and colesevelam (2010) trials failed to show any therapeutic benefit in cholestatic pruritus [29, 30].

### IBAT2330672 trial

The apparent lack of novel drug development in cholestatic pruritus can be attributed partly to incomplete understanding of the complex pathophysiology of the disease. More recent advances in molecular research have identified novel targets for drug development in cholestasis. IBAT inhibitors are novel class of drugs with therapeutic potential in cholestasis. They have been shown be beneficial in cholestasis by the experimental studies and their desired effects on serum and faecal bile acid profile has been proven in healthy people [13, 14].

The BAT117213 study is the first phase 2 multicentre, double-blinded, placebo-controlled crossover trial designed to investigate the safety and efficacy of IBAT inhibitor in PBC patients with pruritus. Unlike the only other phase 2 trial of an IBAT inhibitor drug (LUM001) in PBC (CLARITY study, NCT01904058), the main strength of the BAT117213 study is its crossover design which allows estimating the treatment effect in a smaller number of patients and reduces the between-patient variability and yields a more efficient comparison of treatments than a similar sized parallel group trial. In the BAT117213 study every patient will receive both the study drug and the placebo; therefore each patient will serve as his/her own matched control.

An additional strength of this trial is the utility of patient reported outcomes to measure the treatment response objectively using existing validated tools including the PBC-40 questionnaire and 5-D itch scale as well as a novel, easy-to-use electronic symptom diary. The latter has been specifically developed for this study and it contains morning and evening diaries with questions on itch, fatigue and concentration to comprehensively capture the severity of the symptoms over the preceding 12 h. In addition, the exit interviews conducted at the end of the study provide the opportunity for patients to express their experiences in the study in a semi-structured method that may not have been detected with the more structured patient reported outcomes measures.

The BAT117213 study also provides a unique opportunity to conduct novel, explorative, mechanistic research in patients with cholestatic pruritus. Serum and urine samples obtained during the study will be used to study the metabolic phenotype (metabonomics) of pruritus in PBC by using ^1^H (proton)-nuclear magnetic resonance (NMR) spectroscopy and mass spectrometry (MS). Similarly, using the faecal samples from study patients gut-microbiome studies will be undertaken to study the association between gut microbiota composition and pruritus in PBC. Results of these metabonomic and microbiomic studies are likely to provide more insight into the biology of pruritus in PBC and may identify potential biomarkers for cholestatic pruritus.

The main drawback of this trial is the potential carryover effect (i.e. effect of the treatment from the previous time period may “carry over” on the response to subsequent period) and lack of “washout period” between treatment periods. Carryover effect is a common problem inherent to the cross over study design and may potentially confound direct estimates of treatment effect. Therefore the statistical analysis the data will be assessed for any evidence of carry over and appropriate sensitivity analyses will be performed. To mitigate against the lack of “washout period” the outcome measurements will be restricted to the latter part of each treatment period.

In summary, BAT117213 study is a phase 2 study to evaluate the safety and tolerability of a unique class of drug in treating pruritus in PBC patients and provide novel information about bile acids and metabolic changes and gut microbiome profile in cholestatic pruritus. The results from this trial will inform the trial design of future development phase of the IBAT inhibitor drug.

## Abbreviations

ABPI, association of the British Pharmaceutical Industry; AE, adverse event; ALT: alanine transaminase; ASBT, apical sodium dependent bile acid transporter; AUC, area under the curve; BA, bile acid; C4, 7-alpha-hydroxy-4-cholesten-3-one; CI, confidence interval; CYP, cytochrome P450; ECG, electrocardiogram; eCRF, electronic case report form; ePRO, electronic patient reported outcome; GCP, good clinical practice; GI, gastrointestinal; GSK, GlaxoSmithKline; GUDCA, glyco ursodeoxycholic acid; IBAT, Ileal bile acid transporter; LT, liver transplantation; MS, mass spectrometry; NHS, National Health Service (of the UK); NMR, nuclear magnetic resonance; NRS, numerical rating scale; PBC, primary biliary cholangitis (previously cirrhosis); PGX, pharmacogenetics; PK, pharmacokinetics; PRO, patient reported outcome; RAMOS, randomisation and medication ordering system; RCT, randomised controlled trial; SAE, serious adverse event; SD, standard deviation; TUDCA, tauro ursodeoxycholic acid; UDCA, ursodeoxycholic acid; ULN, upper limit of normal.
